# Self- and cross-attention accurately predicts metabolite–protein interactions

**DOI:** 10.1093/nargab/lqad008

**Published:** 2023-01-31

**Authors:** Pedro Alonso Campana, Zoran Nikoloski

**Affiliations:** Machine Learning, Department of Computer Science, University of Potsdam, 14476 Potsdam, Germany; Bioinformatics, Institute of Biochemistry and Biology, University of Potsdam, 14476 Potsdam, Germany; Bioinformatics, Institute of Biochemistry and Biology, University of Potsdam, 14476 Potsdam, Germany; Systems Biology and Mathematical Modeling, Max Planck Institute of Molecular Plant Physiology, 14476 Potsdam, Germany

## Abstract

Metabolites regulate activity of proteins and thereby affect cellular processes in all organisms. Despite extensive efforts to catalogue the metabolite–protein interactome in different organisms by employing experimental and computational approaches, the coverage of such interactions remains fragmented, particularly for eukaryotes. Here, we make use of two most comprehensive collections, BioSnap and STITCH, of metabolite–protein interactions from seven eukaryotes as gold standards to train a deep learning model that relies on self- and cross-attention over protein sequences. This innovative protein-centric approach results in interaction-specific features derived from protein sequence alone. In addition, we designed and assessed a first double-blind evaluation protocol for metabolite–protein interactions, demonstrating the generalizability of the model. Our results indicated that the excellent performance of the proposed model over simpler alternatives and randomized baselines is due to the local and global features generated by the attention mechanisms. As a results, the predictions from the deep learning model provide a valuable resource for studying metabolite–protein interactions in eukaryotes.

## INTRODUCTION

Metabolites, as low mass compounds produced through metabolic processes, are integral parts of cells, tissues, organs and biological fluids. In addition to serving as energy sources and raw materials for organisms, metabolites bind to proteins via covalent and non-covalent interactions (1–3). These metabolite–protein interactions (MPIs) regulate cellular processes by activating or repressing the activity of proteins involved in diverse cellular processes (e.g. signal transduction and metabolic reactions (4)) and are key to the integration of metabolism and transcription (5). Therefore, characterizing the entirety of MPIs in different cellular systems is of great importance for deepening our understanding of life (6).

The characterization of MPIs consists of resolving three problems: (i) determining whether or not there is an interaction between a metabolite and a protein, (ii) quantifying the binding affinity of the metabolite to the protein and (iii) specification of metabolite–protein interaction sites. All of these problems can be addressed by *in vivo*, *in vitro*, and *in silico* approaches; however, most of these approaches are resource-intensive and allow us to explore only a fraction of MPIs at a time (6). Machine learning has provided flexible and powerful *in silico* frameworks to predict and further analyse MPIs (7,8). These frameworks are considerably less resource intensive than docking studies, even with a full access to protein structures (9). To this end, supervised machine learning approaches can make predictions when only a limited set of labelled data instances exists, as is the case for MPIs (10).

All machine learning frameworks for prediction of MPIs require representations of the input protein and metabolite pairs. This is achieved by: (i) using input protein features (e.g. protein-level global descriptors and amino acid-level descriptors) and compound features (e.g. global chemical features and/or fingerprints encoding the different substructures found in the metabolite) (7,11) or (ii) relying on protein and compound representations learned by deep learning (12,13). These machine learning frameworks usually cast the MPI prediction problem in a classification or regression setting. Supervised machine learning frameworks also require access to reliable instances of non-interacting metabolite–protein pairs. Finally, the generalizability of the model can only be reliably assessed in the double-blind setting in which the model is tested on metabolites and proteins that have not been seen in the training of the model.

Sequence-based deep learning methods represent proteins as a series of amino acids, and apply different techniques, including attention mechanisms (14), with the objective to obtain feature-rich representations. This is done by associating numerical features to each sequence element (usually referred as *tokens* in the deep learning community) or subsets of sequence elements. In this context, for the closely related problem of drug–target interactions, self-attention has been applied over the individual amino acids, where each of them learned a numerical representation (15), or over different representations obtained from the sequences, such as substructures (12) or convolutions of the amino acids (16).

Although successful, self-attention cannot generate interaction-specific features, rendering the subsequent use of features generated by this method impractical. To address this issue, cross-attention (known as joint-attention) has been applied to: (i) concatenated features (17) or (ii) as a pooling strategy based on attention matrices from sequential representations of both metabolites and proteins (18). The utilization of attention for strictly sequential representations, either cannot ensure the generation of interaction-specific features or requires pooling a matrix of attention weights to let the sequence of one element attend over the sequence of the other interacting element.

In contrast, here we rely on self-attention over the amino acid tokens; in addition, we use cross-attention between the non-linearly projected compound features and amino acid tokens to pool the protein sequence. This protein-centric approach results in interaction-specific features derived from the protein sequence alone. The presented approach relies on prior knowledge about MPIs for eukaryotic model organisms. Two publicly available databases, BioSnap (19) and STITCH (20), were preprocessed and used as ground-truth to train a classifier that distinguishes interacting from non-interacting metabolite–protein pairs. These large databases of MPIs cover different protein families, facilitating the design of a double-blind evaluation protocol that has only been attempted in few drug-target prediction studies with limited success (21–23). Finally, we briefly investigate the interpretability of the approach along with a comparison of predictions of MPIs for eukaryotes against well-studied examples from prokaryotes.

## MATERIALS AND METHODS

### Data

Data on MPIs were obtained from two sources: BioSnap (19) and STITCH (20). Biosnap contains 130323 positive interactions for 1774 metabolites and 7716 proteins. From the STITCH database, all entries with a total score above 600 from seven eukaryotes, namely, *Saccharomyces ceverivisiae*, *Arabidopsis thaliana*, *Oryza sativa*, *Danio rerio*, *Caenorhabditis elegans*, *Mus musculus* and *Homo sapiens* were selected. The data on MPIs obtained from STITCH contained in total 3 791 975 interactions for 40 486 proteins and 119 502 compounds, and during training and evaluation. In most of our computational experiments, the data from the different organisms were employed jointly; as a result, the model was trained and evaluated processing all data points without the use of any additional indicator variables.

Furthermore, two different data randomization strategies were employed to generate corrupted versions of the BioSnap data set that serve as additional baselines: (i) randomization of the interacting pairs, by randomly matching the metabolites and proteins found in the true positive set (while respecting their frequencies), and (ii) randomization of the features associated to each identifier (STRING ids for the case of proteins and PubChem CIDs for the case of metabolites).

### Data partitioning to address data leakage

Given the size of the BioSnap data, the metabolite–protein pairs were split using a lenient strategy, where no pair was shared between training and validation data; however, in the lenient strategy, some metabolites and proteins appear both in the training and validation data. In contrast, the STITCH data, given the large number of entries, allowed us to perform a double-blind split, where a fraction of both proteins and metabolites were used as validation data, and the training data contained no pairs containing either of the metabolites and proteins. This results in partitioning of the STITCH MPIs into four sets (or folds): training, protein-blind (where proteins are unseen during training) , metabolite-blind (where the metabolites are not part of the training data ), and double-blind (where neither the metabolites, nor the proteins are found in the training data). More specifically, 21 729 compounds and 8776 proteins from STITCH were used as validation data, resulting in a double-blind partition consisting of 98 861 entries, a metabolite-blind partition with 613 460 interacting pairs and a protein-blind partition containing 506 308 metabolite–protein interactions. To the best of our knowledge double-blind evaluations is rarely performed when evaluating models for drug-target interactions (for exceptions, please, see (21–23)), and, generally, models evaluated under this setting show much lower performance.

Different proteins and metabolites can have a high degree of structural homology, which leads to data leakage in the context of model training and validation. This issue is rarely addressed in the literature, and the exception employed a lenient split setting (24). To avoid possible data leakage under the double-blind setting in our experiments, first metabolites represented by the same fingerprint and proteins represented by the same sequence were collapsed. Second, metabolites and proteins were partitioned into clusters of similar molecules. To this end, the Euclidean distance was used as a distance measure with the PubChem fingerprints, for pairs of metabolites, and with the number of different amino acid residues, for pairs of proteins. Then, if the distance between two species was below a given threshold, they were assigned to the same cluster. To avoid clustering of all small species in the same group, different thresholds were used depending linearly on the sequence length (from 7 used for the shortest protein to 29 used for the largest protein) and the compound mass (from 1.2 to 5). Maximal and minimal thresholds were manually selected, trying to find a partitioning of the species resulting in a moderate number of clusters with proteins or metabolites of diverse sizes. The use of automatic clustering strategies (e.g. K-means), presented the same problems regarding the Euclidean distance between pairs of different sizes; furthermore, it would require the specification of a fixed number of clusters. The use of protein and/or metabolites families, or other partition strategies based on hierarchical classifications of the compounds based on their structural similarities would be the preferred choice; however, the partition of the data set using this strategy was not possible due to the big number of proteins with no associated family. It was also not possible to use other similarity measures insensitive to differences in size (e.g. Jaccard coefficient), since the features contained a combination of continuous and binary features. The use of Euclidean distance as a distance measure is therefore justified.

### Featurizing metabolites and proteins

Each metabolite was featurized using all available features in the PubChem-API (25). These included global properties of the metabolite, such as: molecular weight, solubility, total polar surface area, complexity or volume. Further, they also comprised counts for different entities related to the reactivity and physico-chemical properties, such as number of feasible hydrogen bond donors, acceptors, rotatable bonds, anions or aromatic rings, and the 2D fingerprint, which encoded the presence, absence and counts of different elements and substructures, encoding in total 880 features. Details about these features can be found in PubChem.

The fingerprint was decoded to binary, discrete and continuous features, then non-informative features (where >99% of the instances display the same value) were discarded, each continuous feature was scaled to the range of [ −3, 3], and for each feature missing values were replaced by the mean, and encoded as separate binary variables. This resulted in 630 metabolite features for the metabolites present in BioSnap and 622 for the case of STITCH. Proteins were represented by their sequence, obtained from UniProt. Sequences of length larger than 1022 amino acids were discarded, and each of the remaining sequences was one-hot encoded, including special tokens for the beginning and the end of the sequence. The amino acids of each sequence were then embedded using 128 chemical features obtained from AAindex (26), and each embedding included positional information using positional encoding (all positions were considered informative, and no features were discarded) (27).

### Generation of negative instances

Using a strategy similar to (28), for each of the four partitions (i.e. training, protein-blind, metabolite-blind and double-blind), a set of negative instances was generating by randomly pairing the metabolites and proteins species found in the positive sets. Due to the sparsity of the data, we expect the probability of selecting a genuine positive pair by chance to be low. Nevertheless, to address the presence of false negatives in the data, if a pair was already present in the positive set, the negative instance was discarded. This was performed to ensure that the prediction for each species is zero-centered, and that the relative frequencies of the different metabolites and proteins do not alter the predicted probabilities. Note that this strategy may result in random models whose performance is higher than expected by chance, specially if the number of pairs is low (since a pair might appear more than once in the data).

Finally, using the aforementioned features, the prediction of MPIs was cast as a classification problem, and different models were trained and evaluated on each of the data sets.

### Models

**Figure 1.**
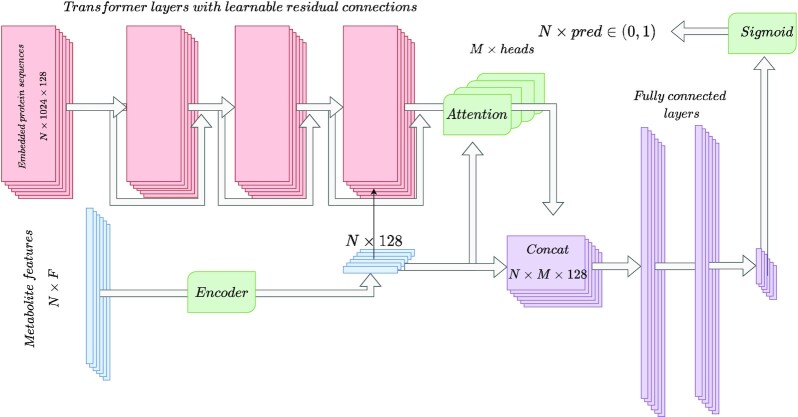
Outline of the architecture for learning of metabolite–protein interactions. Red boxes represent protein data, blue, metabolite data, purple, joint data, and green boxes denote the mappings. A series of transformer encoder layers with residual connections generate an embedded representation of N protein sequences, each amino acid represented by a vector of 128 chemical properties. A fully-connected encoder generates a representation of size 128 for each of *N* metabolites. A multi-head attention pooling module generates pooled representations of the N sequences based on the embeddings of the sequence and the metabolite features. These representations and the encoded metabolite features are concatenated and flattened. A series of fully-connected layers with residual connections generate a single scalar for each pair, which is finally mapped to the (0, 1) range, and interpreted as a probability that the metabolite and protein interact.

We used the same architecture for both gold standards of MPIs. The architecture is composed of a protein sequence embedding module, a compound embedding module, a sequence pooling module and a fully-connected network (see Figure 1). The protein sequence embedding module used a series of Transformer layers (27) with learnable residual connections to generate more informative embeddings of dimension 128. The three Transformer layers were pretrained to predict masked amino acids of the sequence in a similar fashion to how multilayer transformer language models were trained to predict masked words in sentences (29). This kind of models also guided the choice of using a series of stacked Transformer layers, but a lower number (i.e. three) was used to reduce the number of parameters and the computation time. For the model trained on the STITCH data set, the hyperparameters were selected using BOHB (30), where the optimized metric was the geometric mean of the AUC for the three different partitions (double-, protein-, and metabolite- blinds). Note that this leads potentially to data leakage, as no separate train, test and validation folds were employed, but the double-blind partition renders the creation of three folds infeasible, and the large size of the validation set ($\sim \frac{1}{2}$ of the data) makes overfitting unlikely. For the models trained using BioSnap and the baselines, the same hyperparameters were employed. This resulted in an architecture with 32 attention heads in the pooling module, 5 residual blocks with a hidden dimension of 2048 and a dropout probability of 0.33, a learning rate of 3.1 × 10^−5^ and a weight decay of 7.2 × 10^−7^.

Further, the metabolite embedding module contained a batch normalization layer along with a series of linear layers and non-linear activation functions. It maps each metabolite to a lower dimensional space ($\mathcal {R}^{128}$), and can be regarded as a learnable dimensionality reduction module that could help in reducing the effect of correlated features on model performance.

The sequence pooling module used multi-head cross-attention to pool the sequence based on the transformed features of each amino acid and the transformed embedded features of each metabolite. For each head, first, both the metabolite and element-wise encoded protein features were projected to a common attention space:

$$\begin{equation*} H_{met_i} = \phi _{met}(X_{met_i}) \end{equation*}$$

$$\begin{equation*} H_{prot_{jk}} = \phi _{prot}(X_{prot_{jk}}) \end{equation*}$$

where ϕ_*met*_ and ϕ_*prot*_ are neural networks consisting of two linear layers with a non-linear function between them (e.g. rectified liner unit (31)).

Second, the scaled dot-product is used to generate a similarity measure between the projected features of the metabolite and the projected features of each amino acid:

$$\begin{equation*} \theta (met_i, prot_{jk}) = \frac{H_{met_i} \cdot H_{prot_{jk}}}{\sqrt{N}}, \end{equation*}$$

where *N* denotes the sequence length (in our experiments *N* = 1024).

Third, for each protein sequence, the similarity measures are transformed into attention weights using a softmax function:

$$\begin{equation*} \alpha _{ijx} = \frac{e^{\theta (met_i, prot_{jx})}}{\sum _{k=1}^{N} e^{\theta (met_i, prot_{jk})}} \end{equation*}$$

Then, the transformed sequence features are pooled using these attention weights and interaction-specific features are generated, as follows:

$$\begin{equation*} Z_{met_i, prot_{j}} = \sum _{k=1}^{N}\alpha _{ijk} H_{prot_{jk}}. \end{equation*}$$

Finally, the different sequence representations generated by each head are mapped to a lower dimension and are concatenated, generating a feature-rich representation for each interaction:

$$\begin{equation*} H_{met_i, prot_{j}} = \biggm | \biggm | _{h=1}^M\phi _{head}^h(Z_{met_i, prot_{j}}^h), \end{equation*}$$

where $\biggm | \biggm |$ denotes concatenation, *M*, the number of heads (*M* = 16 in our experiments), and ϕ_*head*_ denotes a learnable linear layer that maps the attention space of size *S* to *S*//*M*.

Additionally, architectures were generated where the transformer layers were replaced by the identity mapping, and where the attention module was replaced by average pooling. This aimed at providing simpler alternative models to evaluate the individual and joined effect of using attention as a modelling strategy.

The features generated by the pooling module were in turn transformed by a series of fully-connected layers with non-linear activation and residual connections. The multidimensional feature space was then collapsed to a single scalar thanks to a linear layer and are mapped to the (0, 1) range using a sigmoid function.

Using PyTorch (32), all models, including the different baselines, randomization strategies and data sets, were trained for 40 epochs using Adam (33) to minimize the binary cross-entropy between the observed and predicted values. Transformers (27) requires the computation of a $\mathcal {R}^{n \times n}$ matrix, where *n* represents the length of the sequence. This constitutes a computational bottleneck with $\mathcal {O}(n^2)$ time and memory complexity. Due to this fact and the length of protein sequences, training was time-intensive; therefore, a low number of epochs (40) was chosen for model training. The learning rate was effectively scheduled by progressively using a larger batch size while the weight decay was proportionally decreased, keeping the ratio between them constant, as suggested in (34).

## RESULTS

### Model performance trained on the BioSnap data set

First, we used the BioSnap data set, consisting of 130 323 positive interactions for 1774 metabolites and 7716 proteins, to evaluate the performance of four models. Two models used a transformer and two directly employed of the embeddings of the amino acid properties. In addition, two models pooled the sequence using attention pooling and the other two used average pooling. The four models were trained on the original data and permuted data. The performance of the models was quantified using the Area under the ROC curve (AUCROC) and accuracy (accuracy using 0.5 as the decision threshold). Note that the model that made use of transformer and attention pooling corresponds to the proposed architecture on Figure 1, consisting of a protein sequence embedding module, a compound embedding module, a sequence pooling module and a fully-connected network (see Materials and Methods).

**Figure 2.**
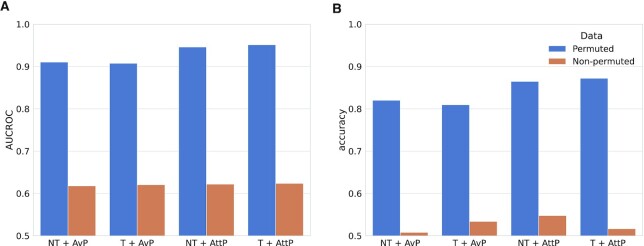
Performance of four models on the BioSnap data and its randomization. Four different models were trained during 40 epochs. Two models use a transformer (T) and two make direct use of the amino acid properties as embeddings (NT). Two models pool the sequence using attention pooling (AttP), and other two use average pooling (AvP). These models were trained on the original data (blue) and permuted data (orange).

The comparative analyses showed that models using the attention pooling module yielded the best performance with the non-permuted data (Figure 2). Further, we found that the four considered models showed higher performance in comparison to the randomized baselines with respect to both measures. The simpler alternatives yielded a surprisingly high performance: The simplest model showed an AUCROC of 0.912 and an accuracy of 0.820, the model using only the transformer showed a slightly lower performance (AUCROC = 0.908, accuracy = 0.810), and the model using the attention-pooling showed the highest performance among the contenders (AUCROC = 0.947, accuracy = 0.865). The performance of the model using all the components was at least as high as that of the simpler models (AUCROC = 0.948, accuracy = 0.872).

We also found that the performance of the randomized baselines was slightly higher than expected by chance for both performance measures. This observation can be readily explained by the data randomization process and the characteristics of the data set. More specifically, BioSnap is relatively dense and, as a result, the permutation of metabolite–protein leads to positive duplicate entries. Since entries found in the set of positive instances are discarded during the creation of the set of negative instances (see Materials and Methods), duplicate entries are exclusively positive and can be split during the train-test validation split. This artefact cannot contribute to the performance measures using the non-permuted data, because these data do not contain duplicates. Additional performance measures for the randomized baselines can be found in Supplementary Table S2 and Supplementary Figure S2.

Given the high performance of the simpler models, as a sanity check we also trained a simple logistic regression model using the average pooling and encoded metabolite features. The performance of this model was close to random (the binary cross-entropy was 0.659, close to the expected performance of a random classifier (ln(2) ∼ 0.693)) and AUCROC = 0.621. Together, these findings demonstrated that the proposed model outperforms simpler, contending alternatives and can next be used to test its generalizability.

### Model performance trained on the STITCH data set

**Figure 3.**
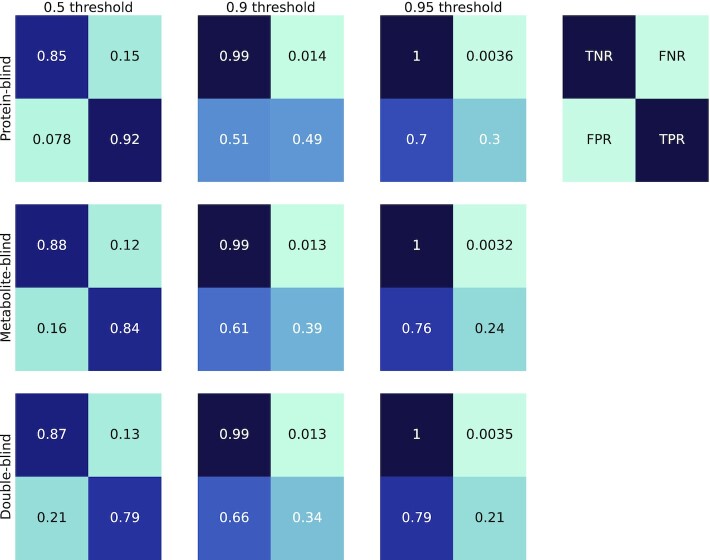
Confusion matrices for the different splits on the STITCH data set. The performance of the model on the three different sets of unseen pairs, i.e. protein-blind, metabolite-blind, and double-blind fold, was compared using three thresholds for positive classification, i.e. 0.5, 0.9 and 0.95. The model generalizes for the three different folds. TPR stands for True Positive Rate, FPR for False Positive Rate, TNR for True Negative Rate, and FNR for False Negative Rate.

The model resulting from the training based on the STITCH data set is the main object of our interest, as it represents the biggest source of available knowledge about metabolite–protein interactions under a single model. Due to the high computational costs of training, only the full model (Figure 1) was evaluated on this data set for the protein-blind, metabolite-blind, and double-blind folds. We inspected the performance of the model in terms of the true positive, true negative, false positive, and false negative rates along with the specificity and sensitivity for the different folds (Figure 3). These performance measures were chosen as they allow a more comprehensive evaluation of model performance. More specifically, for all possible interaction-pairs, a very large class imbalance is to be expected, of which a big fraction is composed of negative instances. For this reason, performance measures such as AUCROC and accuracy might me misleading. The accuracy using a 0.5 classification threshold, *F*1 scores and AUCROC for the different partitions are shown in Table 1. As expected, for all metrics the model showed the lowest performance under the double-blind partition (AUCROC = 0.915), followed by the metabolite-blind partition (AUCROC = 0.936), and protein-blind partition (AUC = 0.953).

**Table 1.** Performance metrics for the different partitions. The table provides three performance measures: accuracy, F1 score, and AUROC for the metabolite-blind, protein-blind, and double-blind partition

	Accuracy	*F*1 score	AUCROC
Metabolite-blind	0.860	0.865	0.936
Protein-blind	0.885	0.898	0.953
Double-blind	0.829	0.829	0.915

Since the expected number of true negatives is much higher than the expected number of true positives, we selected three threshold values of at least 0.5 in these evaluations (i.e. 0.5, 0.9 and 0.95). As expected, employing a higher classification threshold increased the specificity and decreased the sensitivity of the models. Although all folds showed good performance, under the protein-blind setting the model showed the highest sensitivity (0.92, 0.49 and 0.3, respectively) using the three different thresholds; moreover, for the metabolite-blind fold the model exhibited the highest specificity. Lastly, for the double-blind fold, the model showed an intermediate specificity (0.87 with a 0.5 threshold) and the lowest sensitivity (0.79 using the 0.5 threshold).

The model was also evaluated using data from species not used during training, to demonstrate that the performance was still better than random, but it was lower. For instance, under the double-blind setting, for the prokaryotic model organisms *Escherichia coli*, AUCROC was 0.62, while for the microalga *Chlamydomonas reinhardtii*, AUCROC was 0.67 (see Supplementary Table S1).

Together, these results indicated that the model exhibited very good generalizability for the species used for training the model, warranting the usage of the predicted MPIs for planning validation experiments. However, usage of the models for other organisms warrants caution. The model trained using all of the data obtained from STITCH is provided as a tool in https://github.com/alonsocampana/scampi. Using this tool, one can readily predict interaction probability for metabolite–protein pairs specified by PubChem compound IDs and protein sequence as inputs.

### Tasks performed by attention heads in the double-blind fold

**Figure 4.**
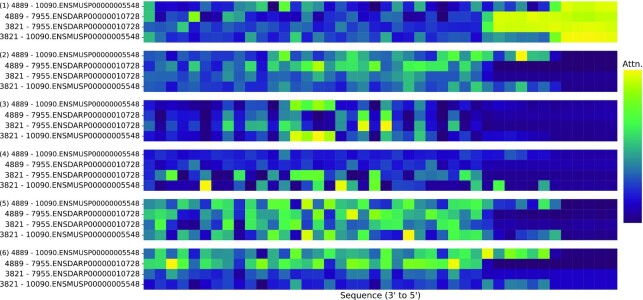
Attention over the protein sequence for six heads. Four positive metabolite–protein pairs (rows), identified by their compound ids and STRING ids and unseen during training were selected and the attention over the different amino acids found in each protein sequence was analyzed. For six different attention heads, the attention score associated to each amino acid was summed every eight positions, to allow a more clear visualization of the sequence. The more yellow hue indicates higher attention, and correspond to sequence regions with a higher importance in the pooled representation generated by that head.

The attention weights generated by the STITCH model were analysed for pairs obtained from the double-blind fold. As can be seen in Figure 4, different heads have learnt to perform different tasks, generating complex features by assigning high attention weights to different elements of the sequence. For instance, the first head on Figure 4 attended over the padding, and generates features related to the length of the sequence, while the second head on Figure 4 pooled mostly average features. Further, the third and fifth heads attended over particular positions independently of the metabolite, while the fourth and sixth head focused attention on concrete spots on a metabolite-dependent way. Therefore, the usage of attention pooling facilitated the generation of both local and global features important for boosting the performance of the model and its generalizability.

### Predictions of MPIs for *A. thaliana*

To better understand the insights provided by the model, we next used it to predict the probability of interactions for pairs unseen during training.

**Figure 5.**
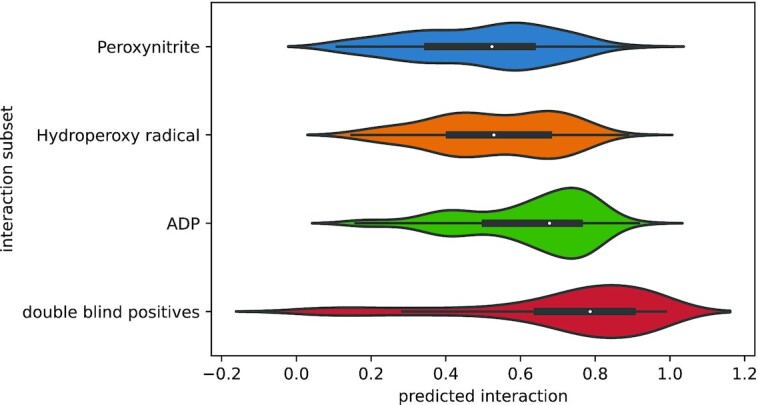
Distributions for the predictions using *A. thaliana* proteins and four subsets of interactions The model was used to generate predictions between 123 proteins unseen during training and four sets of metabolites: Double-blind positives, two reactive oxygen species and ADP. The Y axis shows the predicted interaction probability for each pair, and each violin plot shows the distribution of predicted interaction probabilities for a subset of interaction (shown in the X axis).

In one experiment the model was used to generate predictions for the 200 double-blind *A. thaliana* instances with the highest STITCH score. This resulted in a set containing 123 proteins and 35 metabolites. The same set of proteins was used to generate predictions for two reactive oxygen species (Hydroperoxy and Peroxynitrite), expected to show interactions with many proteins, and ADP, expected to be involved in more specific interactions (Figure 5). The reactive oxygen species displayed predicted interaction distributions, apparently, close to 0.5-centered distribution (Hydroperoxy: $\bar{p} = 0.531 \pm 0.012$, Peroxynitrite: $\bar{p} = 0.493 \pm 0.013$). For ADP, this distribution displayed a higher mean ($\bar{p} = 0.625 \pm 0.011$). Further, the interactions found in the gold standard showed the highest average interaction probability ($\bar{p} = 0.718 \pm 0.018$), and also the highest variance, where, interestingly, some interactions were assigned probabilities close to 0.

The model was also used to generate interaction predictions for nine metabolites, experimentally tested in *E. coli* (35), including: 2-oxoglutaric acid, Phosphoenolpyruvate, Adenosine tri- and diphosphate (ATP, ADP), l-phenylalanine, Succinate, Malate, Glutamate and Aspartate. The predictions were generated for 214 *A. thaliana* proteins not seen during model training. These proteins belonged to three different families, i.e. kinases (112), hydrolases (52), and peptidases (50). We found that the nine metabolites exhibited different distributions for the probabilities of interaction (Supplementary Figure S1); more details about the statistically significant differences between these distributions can be found in Supplementary Figure S1. The 2-oxoglutaric acid showed the smallest average probability (0.47) for interaction, while succinate exhibited the highest (0.62) over the 214 proteins. We next tested if there were differences in the average probability of interaction for one metabolite between protein families using two-sided pairwise *t*-test and Benjamini-Hochberg for multiple hypotheses testing correction. For phosphoenolpyruvate, succinate, glutamate and aspartate, hydrolases showed the highest interaction probability. For 2-oxoglutarate, ATP and ADP, the interaction probabilities were larger for the kinases, but the differences with the hydrolases were not statistically significant.

Interestingly, we found that there were proteins that showed low interaction probabilities for all nine metabolites. These proteins included AT1G08720, a kinase related to salicylic acid regulation, or AT1G79690, a homolog of the Nudix hydrolase. Further, the hydrolases and the kinases tended to have higher interaction probabilities with all nine tested metabolites. Typical examples of predicted interacting pairs included AT1G34430 (part of pyruvate dehydrogenase complex) and phosphoenolpyruvate (predicted interaction probability 0.947), or AT1G23460 (an hydrolase involved in lignin biosynthetic process) and Glutamate (predicted interaction probability 0.931). The interaction probability of arbitrary metabolite–protein pairs can be readily investigated in detail by the provided tool. More details about the distribution of predictions for each metabolite and protein family can be found in Supplementary Figure 1.

## DISCUSSION

We presented and carefully evaluated a deep learning model that is able to predict MPIs for unseen proteins and metabolites with high sensitivity (0.79) and specificity (0.87). We further showed that the proposed architecture performed better under a lenient split (AUCROC = 0.948, accuracy = 0.872), compared to randomized and other baselines, including simplified versions of the model. Surprisingly, the use of an architecture without a Transformer showed a very similar performance compared to the complete architecture. This could be due to the relatively small size of the BioSnap data set on which the different models were tested, and is in line with observations from other fields, such as Natural Language Processing, demonstrating that Transformers require huge data sets to outperform other modelling strategies (36). In general, simpler deep learning models have also shown high performance, but a logistic regression model trained on the same features performed poorly. This observation suggested that the interactions between some of the features are responsible for the model predictions. Different data randomization strategies showed that the model exploits mainly the similarity between the protein sequences to generate its predictions (since randomization of the protein sequences decreases performance by a high margin); the similarities between the metabolites based on their features is of secondary importance (since the permutation of the metabolite features also decreases performance, but to a smaller degree). It was not possible to compare the performance of the different models using hypothesis testing, because bootstrap and cross-validation are incompatible with a model of this size.

We also analyzed the attention mechanism learnt by the model, and found the different attention heads performed different tasks. This observation highlights the ability and flexibility of this model to generate rich features from protein sequences. Metabolite fingerprints were used as descriptors, because they provide one-dimensional representations for each molecule and do not require: (i) additional pooling to generate global metabolite-wise features or (ii) additional layers for feature extraction, such as Transformers or Convolutional Networks, used with sequential data. However, they present two drawbacks: In the case of drug-target interactions and general molecular modelling tasks, it was shown how feature-rich representations, such as SMILES in the form of strings or graphs have shown better performance (37). Furthermore these representations provide additional interpretation possibilities. Future research may replace fingerprints by the mentioned representations.

The comparison between the predictions for different sets of metabolites, showed that positive, well-known interactions display higher interaction probabilities, even when compared against random interactions involving a frequent and specific interactor, such as ADP. Additionally, it showed how unspecific interactions, such as those involving oxygen reactive species, display average interaction probabilities close to 0.5, i.e. the model shows lowest certainty about these interactions belonging to the positive or negative classes. Finally, the predictions generated for nine compounds and 214 *A. thaliana* proteins showed that the model predicted statistically significance differences between the average interaction probabilities for some protein families and metabolites (Supplementary Figure S1). These results are contrast with those reported for *E. coli*, where ATP and ADP were found to act as ubiquitous interactors across all tested proteins (35). The differences may be due to the usage of eukaryote-specific gold standards (ATP was ranked the 15th more frequent interacting metabolite, with 5729 positive interactions, and was used for training, ADP 18th with 4993 positive interactions, was used for validation) or the bias due to the preferential study of particular proteins and/or metabolites.

While there have been other attempts to generate deep learning models using similar inputs (18,37–40), they focus on predicting drug-target interactions. This focus differs from the prediction of metabolite–protein interactions for two reasons: First, the data sets used contain a considerably smaller number of proteins, e.g. the Davis data set with 30056 interactions for 442 compounds and 68 proteins, KIBA with 229 proteins, 2111 compounds and 118254 interactions, or the DTIminer data set with 4503 drugs, 2182 proteins and 15 138 drug-target interactions. Furthermore, these data sets contain continuous variables and the models learn a regression problem. In contrast, here we trained and evaluated models on BioSnap and the subset of STITCH with 7716 and 40 486 different proteins, respectively, that belong to different protein families. Second, the evaluations performed in the context of drug-target interactions lack a detailed double-blind evaluation protocol, likely related to the possibility on only performing lenient split evaluations and do not prevent data leakage. For these reasons, it is not possible or meaningful to compare the performance of the existing model to our approach.

As a result, our model provides novel means to predict MPIs at a proteome-wide level. The demonstrated generalizability of the proposed model along with careful investigations with two large gold standards render the predictions a valuable resource for further investigation and experimental validation of the MPIs in eukaryotes.

## DATA AVAILABILITY

Driver code that can be used to generate predictions for metabolite–protein interactions using the trained models and to train the models from scratch can be found in https://github.com/alonsocampana/scampi, and in https://zenodo.org/record/7457367#.Y6BLTafMLJU the raw data from STITCH used for the double-blind experiments can be downloaded from http://stitch.embl.de/, the raw data from BioSnap used for the lenient split evaluation and permutation experiments is hosted under http://snap.stanford.edu/biodata/datasets/10016/10016-ChG-InterDecagon.html. The protein sequences and cross-references for the STITCH database can be obtained from https://www.uniprot.org/. Amino acid features can be found in https://www.genome.jp/aaindex/, and metabolite features can be downloaded from https://pubchem.ncbi.nlm.nih.gov/.

## Supplementary Material

lqad008_Supplemental_File
