# Supplementary material for: Self- and cross-attention accurately predicts metabolite–protein interactions
Source: NAR Genom Bioinform. 2023 Jan 31;5(1):lqad008. doi: 10.1093/nargab/lqad008 (PMC9887643; doi:10.1093/nargab/lqad008)
Supplement: lqad008_Supplemental_File [file lqad008_supplemental_file.pdf]

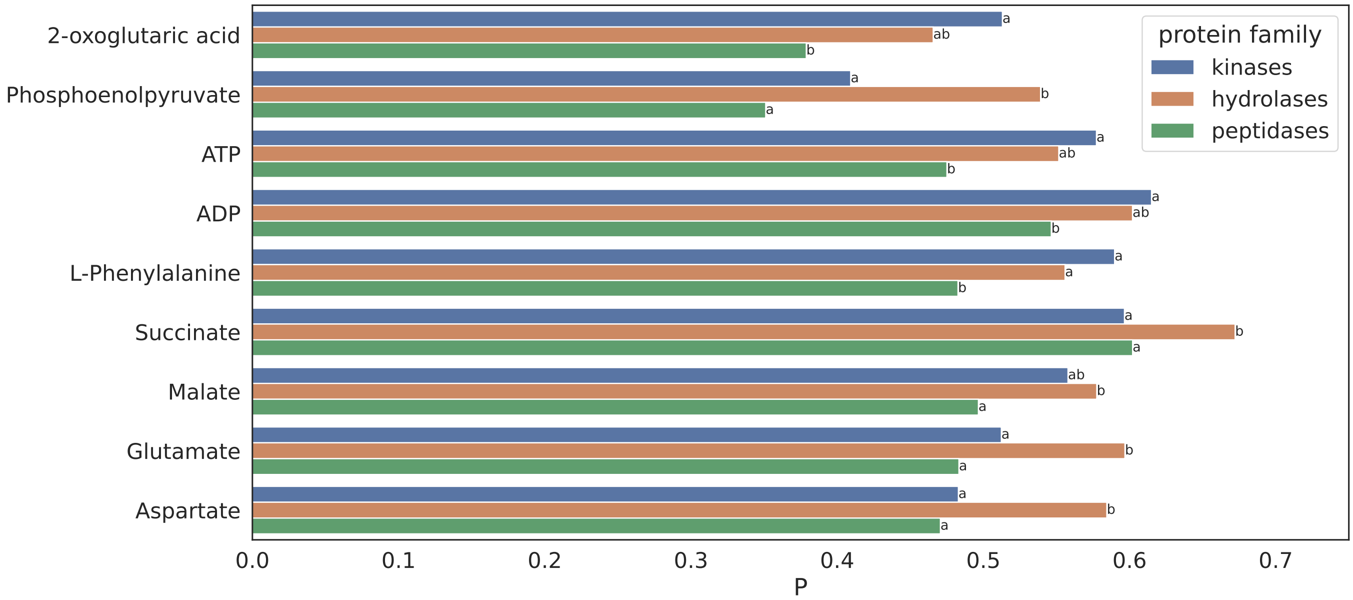

Supplementary Figure 1. Predictions using *A. thaliana* proteins and nine metabolites The model was used to generate predictions between 214 proteins unseen during training belonging to three families (kinases, peptidases and proteases), and nine metabolites (selected from Piazza et al. 2018). The average prediction for each family and metabolite are presented. Differences on between-family means for each metabolite were tested using pairwise t-tests corrected via the Benjamini-Hochberg method($\alpha = 0.05$). If for a given metabolite the difference between family-specific means is statistically significant, they do not share any of the letters above the bars.

	Chlamydomonas double Blind	Chlamydomonas protein Blind	E. coli double Blind	E. coli protein Blind
AUCROC	0.671377	0.710414	0.616733	0.632547
Accuracy	0.612989	0.629970	0.568786	0.559906
F1Score	0.607575	0.712594	0.646156	0.715506
Average precision	0.700773	0.742728	0.638136	0.669094

Supplementary Table 1. Performance of the model using organisms unseen during training The model trained on STITCH data was used to generate predictions for organisms where none of its proteins were present in the training data, *E.coli* and *Chlamydomonas reinhardtii*. Using the methodology for evaluation described in the main text, different performance measures were recorded. Note that *Chlamydomonas reinhardtii* belongs to a division unseen during training *Chlorophyceae* and *E.coli* is a Prokaryote.

FEATURES USED FOR EMBEDDINGS THE AMINO ACIDS

The list of features used for embedding the amino acids was:

alpha-CH chemical shifts, Hydrophobicity index, Signal sequence helical potential, Membrane-buried preference parameters, Conformational parameter of inner helix, Conformational parameter of beta-structure, Conformational parameter of beta-turn, Average flexibility indices, Residue volume, Information value for accessibility; average fraction 35, Information value for accessibility; average fraction 23, Retention coefficient in TFA, Retention coefficient in HFBA, Transfer free energy to surface, Apparent partial specific volume, alpha-NH chemical shifts, Spin-spin coupling constants 3JHalpha-NH, Normalized frequency of alpha-helix, Normalized frequency of extended structure, Steric parameter, Polarizability parameter, Free energy of solution in water, kcal/mole, The Chou-Fasman parameter of the coil conformation, A parameter defined from the residuals obtained from the best correlation of the Chou-Fasman parameter of beta-sheet, The number of atoms in the side chain labelled 1+1, The number of atoms in the side chain labelled 2+1, The number of atoms in the side chain labelled 3+1, The number of bonds in the longest chain, A parameter of charge transfer capability, A parameter of charge transfer donor capability, Average volume of buried residue, Residue accessible surface area in tripeptide, Residue accessible surface area in folded protein, Proportion of residues 95 buried, Proportion of residues 100 buried, Normalized frequency of beta-turn, Normalized frequency of beta-sheet, Normalized frequency of N-terminal helix, Normalized frequency of C-terminal helix, Normalized frequency of N-terminal non helical region, Normalized frequency of C-terminal non helical region, Normalized frequency of N-terminal beta-sheet, Normalized frequency of C-terminal beta-sheet, Normalized frequency of N-terminal non beta region, Normalized frequency of C-terminal non beta region, Frequency of the 1st residue in turn, Frequency of the 2nd residue in turn, Frequency of the 3rd residue in turn, Frequency of the 4th residue in turn, Normalized frequency of the 2nd and 3rd residues in turn, Normalized hydrophobicity scales for alpha-proteins, Normalized hydrophobicity scales for beta-proteins, Normalized hydrophobicity scales for alpha+beta-proteins, Normalized hydrophobicity scales for alpha/beta-proteins, Normalized average hydrophobicity

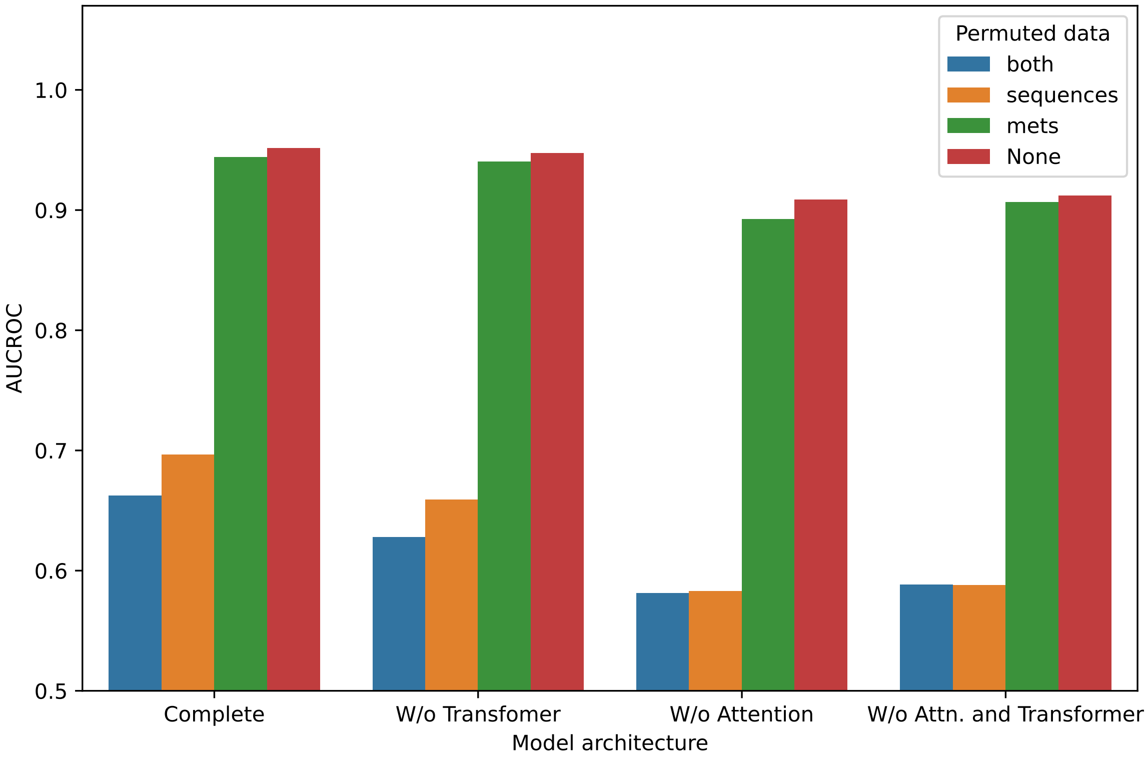

Supplementary Figure 2. Comparison of the different architectures using different feature permutations Using the BioSnap data, the features for the differents proteins and/or metabolites where permuted, the model was trained for 40 epochs, and the performance was recorded using the Area Under the ROC Curve

No attn.	No Trans.	Permuted Proteins	AUCROC	Accuracy	F1Score	AveragePrecision	Precision	Recall
True	True	False	0.910342	0.820087	0.882163	0.934085	0.826538	0.945816
True	False	False	0.907526	0.809563	0.876294	0.933580	0.816320	0.945780
False	True	False	0.945885	0.864741	0.906938	0.961737	0.868643	0.948765
False	False	False	0.951328	0.872014	0.915734	0.964697	0.868621	0.968250
True	True	True	0.617895	0.507983	0.739084	0.738435	0.600164	0.961687
True	False	True	0.620679	0.533807	0.712916	0.740432	0.616639	0.844821
False	True	True	0.621861	0.547710	0.701665	0.744794	0.626362	0.797548
False	False	True	0.623690	0.516654	0.733388	0.743211	0.604877	0.931237

Supplementary Table 2. Detailed performance displayed by the ablated models Additional performance measures are displayed for the setting used in figure 2. No attention corresponds to the ablation of the attention module, No Transformer to the replacement of the transformer encoder by the identity, and Permuted Proteins to the permutation of the protein set found in the positive set, resulting in a set with random interactions.

scales, Partial specific volume, Normalized frequency of middle helix, Normalized frequency of turn, Size, Amino acid composition, Relative mutability, Membrane preference for cytochrome b: MPH89, Average membrane preference: AMP07, Consensus normalized hydrophobicity scale, Solvation free energy, Atom-based hydrophobic moment, Direction of hydrophobic moment, Molecular weight, Melting point, Optical rotation, pK-N, pK-C, Hydrophobic parameter pi, Graph shape index, Smoothed upsilon steric parameter, Normalized van der Waals volume, STERIMOL length of the side chain, STERIMOL minimum width of the side chain, STERIMOL maximum width of the side chain, N.m.r. chemical shift of alpha-carbon, Localized electrical effect, Number of hydrogen bond donors, Number of full nonbonding orbitals, Positive charge, Negative charge, pK-a(RCOOH), Helix-coil equilibrium constant, Helix initiation parameter at posision i-1, Helix initiation parameter at posision i,i+1,i+2, Helix termination parameter at posision j-2,j-1,j, Helix termination parameter at posision j+1, Partition coefficient, Alpha-helix indices, Alpha-helix indices for

alpha-proteins, Alpha-helix indices for beta-proteins, Alpha-helix indices for alpha/beta-proteins, Beta-strand indices, Beta-strand indices for beta-proteins, Beta-strand indices for alpha/beta-proteins, Aperiodic indices, Aperiodic indices for alpha-proteins, Aperiodic indices for beta-proteins, Aperiodic indices for alpha/beta-proteins, Hydrophobicity factor, Composition, Polarity, Volume, Partition energy, Hydration number (Hopfinger, 1971), Cited by Charton-Charton, Hydrophilicity value, Heat capacity, Absolute entropy, Entropy of formation, Normalized relative frequency of alpha-helix, Normalized relative frequency of extended structure, Normalized relative frequency of bend, Normalized relative frequency of bend R, Normalized relative frequency of bend S, Normalized relative frequency of helix end, Normalized relative frequency of double bend, Normalized relative frequency of coil, Average accessible surface area, Percentage of buried residues, Percentage of exposed residues, Ratio of buried and accessible molar fractions, Transfer free energy, Hydrophobicity, pK (-COOH).

FEATURES USED FOR THE METABOLITES

The metabolites were featurized using:

MolecularWeight, XLogP, TPSA, Complexity, Charge, HBondDonorCount, HBondAcceptorCount, RotatableBondCount, HeavyAtomCount, AtomStereoCount, BondStereoCount, CovalentUnitCount, Volume3D, XStericQuadrupole3D, YStericQuadrupole3D, ZStericQuadrupole3D, FeatureAcceptorCount3D, FeatureDonorCount3D, FeatureAnionCount3D, FeatureCationCount3D, FeatureRingCount3D, FeatureHydrophobeCount3D, Fingerprint2D.

A more detailed description of the features included in the Fingerprint2D can be found in `https://web.cse.ohio-state.edu/~zhang.10631/bak/drugreposition/list_fingerprints.pdf`.
